# Supplementary material for: Code sets for respiratory symptoms in electronic health records research: a systematic review protocol
Source: BMJ Open. 2019 Mar 3;9(3):e025965. doi: 10.1136/bmjopen-2018-025965 (PMC6443061; doi:10.1136/bmjopen-2018-025965)
Supplement: Supplementary data [file bmjopen-2018-025965supp001.pdf]

Strategy 463864

#	Database	Search term	Results
1	EMBASE	exp "MEDICAL RECORD"/ OR "ELECTRONIC HEALTH RECORDS"/ OR "ELECTRONIC HEALTHCARE DATABASE"/ OR "ELECTRONIC MEDICAL RECORD SYSTEM"/	211275
2	EMBASE	(EHR OR "Electronic Health Record*" OR ((electr* OR digit* OR computer* OR linked OR administrative OR routine OR personal OR patient OR health OR medical) AND (record* OR database*))).ti,ab,if	932977
3	EMBASE	(1 OR 2)	1012318
4	EMBASE	CODING/ OR exp "PATIENT CODING"/ OR "CODING ALGORITHM"/	26988
5	EMBASE	(code OR codes OR coding OR codelist* OR codeset* OR "value set" OR phenotyp*).ti,ab,if	953908
6	EMBASE	(4 OR 5)	974209
7	EMBASE	exp COUGHING/	104032
8	EMBASE	exp WHEEZING/	23552
9	EMBASE	exp SPUTUM/	22594
10	EMBASE	exp DYSPNEA/ OR exp DYSPNOEA/	143174
11	EMBASE	("respirat* symptom*" OR (respirat* AND symptom*) OR breath* OR dyspn* OR sputum OR phlegm OR cough* OR wheez*).ti,ab,if	375955
12	EMBASE	(7 OR 8 OR 9 OR 10 OR 11)	491214
13	EMBASE	(3 AND 6 AND 12)	1108
14	EMBASE	3 AND 6 AND 12 [DT 1990-2017] [English language]	1005

Strategy 463861

#	Database	Search term	Results
1	Medline	exp "MEDICAL RECORDS"/ OR "HEALTH RECORDS, PERSONAL"/ OR "MEDICAL RECORD LINKAGE"/ OR "MEDICAL RECORDS SYSTEMS, COMPUTERISED"/ OR "ELECTRONIC HEALTH RECORDS"/	134717
2	Medline	(EHR OR "Electronic Health Record*" OR EMR OR "Electronic Medical Record*" OR ((electr* OR digit* OR computer* OR linked OR administrative OR routine OR personal OR patient OR medical OR health) AND (record* OR database*))).ti,ab,af	909067
3	Medline	(1 OR 2)	937921
4	Medline	exp "CLINICAL CODING"/	1753
5	Medline	(code OR codes OR coding OR codelist* OR codeset* OR "value set" OR phenotyp*).ti,ab,af	821866
6	Medline	(4 OR 5)	821866
7	Medline	exp COUGH/	14517
8	Medline	exp "SIGNS AND SYMPTOMS, RESPIRATORY"/	165589
9	Medline	exp DYSPNEA/	19188
10	Medline	exp SPUTUM/	19961
11	Medline	("respirat* symptom*" OR (respirat* AND symptom*) OR breath* OR dyspn* OR sputum OR phlegm OR cough* OR wheez*).ti,ab,af	293228
12	Medline	(7 OR 8 OR 9 OR 10 OR 11)	401014
13	Medline	(3 AND 6 AND 12)	620
14	Medline	3 AND 6 AND 12 [DT 1990-2017] [Languages English]	550
