# Supplementary material for: Code sets for respiratory symptoms in electronic health records research: a systematic review protocol
Source: BMJ Open. 2019 Mar 3;9(3):e025965. doi: 10.1136/bmjopen-2018-025965 (PMC6443061; doi:10.1136/bmjopen-2018-025965)
Supplement: Author's manuscript [file bmjopen-2018-025965.draft_revisions.pdf]

BMJ Open is committed to open peer review. As part of this commitment we make the peer review history of every article we publish publicly available.

When an article is published we post the peer reviewers' comments and the authors' responses online. We also post the versions of the paper that were used during peer review. These are the versions that the peer review comments apply to.

The versions of the paper that follow are the versions that were submitted during the peer review process. They are not the versions of record or the final published versions. They should not be cited or distributed as the published version of this manuscript.

BMJ Open is an open access journal and the full, final, typeset and author-corrected version of record of the manuscript is available on our site with no access controls, subscription charges or pay-per-view fees (http://bmjopen.bmj.com).

If you have any questions on BMJ Open's open peer review process please email info.bmjopen@bmj.com

BMJ Open

Codelists for respiratory symptoms in electronic health records (EHR) research: a systematic review protocol

Journal:	*BMJ Open*
Manuscript ID	bmjopen-2018-025965
Article Type:	Protocol
Date Submitted by the Author:	10-Aug-2018

Complete List of Authors:	Jayatunga, Wikum; University College London, Institute of Health Informatics<br>Stone, Philip; Imperial College London, National Heart and Lung Institute<br>Aldridge, Robert; University College London, Institute of Health Informatics<br>Quint, Jennifer; Imperial College London, Respiratory Epidemiology, Occupational Medicine and Public Health<br>George, Julie; University College London, Institute of Health Informatics

Keywords:	Electronic Health Records, Coding, Clinical Coding, Respiratory, Symptoms

SCHOLARONE™
Manuscripts

For peer review only

Title

Codelists for respiratory symptoms in electronic health records (EHR) research: a systematic review protocol

Authors

Dr Wikum Jayatunga[a], wikumj@gmail.com
Mr Philip Stone[b], p.stone@imperial.ac.uk
Dr Rob Aldridge[a], r.aldridge@ucl.ac.uk
Dr Jenni Quint[b], j.quint@imperial.ac.uk
Dr Julie George[a], j.george@ucl.ac.uk

[a] University College London, Institute of Health Informatics, 222 Euston Road, London NW1 2DA
[b] Imperial College London, National Heart and Lung Institute, 1B Manresa Road, London, SW3 6LR

Corresponding Author
Dr Wikum Jayatunga
Address: 3 Constable Mews, Bromley, BR1 3BF
Email: wikumj@gmail.com
Telephone: 07872959152

For peer review only

Keywords

Electronic Health Records, Coding, Clinical Coding, Respiratory, Symptoms

Wordcount

Word count = 1417
(excluding title page, article summary, abstract, references, figures and declarations)

Abstract

Introduction: Asthma and chronic obstructive pulmonary disease (COPD) are common respiratory conditions which result in significant morbidity worldwide. These conditions are associated with a range of non-specific symptoms, which in themselves are a target for health research. Such research is increasingly being conducted using electronic health records (EHRs), but computable phenotype definitions, in the form of codelists, are required to extract structured data from these large routine databases in a systematic and reproducible way. The aim of this protocol is to specify a systematic review to identify codelists for respiratory symptoms in electronic health records research.

Methods and Analysis: MEDLINE and EMBASE databases will be searched using terms relating to electronic health records, respiratory symptoms and use of codelists. The search will cover all English-language studies in these databases between January 1990 and January 2018. Two reviewers will independently screen identified studies for inclusion, and key data will be extracted into a uniform table, facilitating cross-comparison of codes used. Disagreements between the reviewers will be adjudicated by a third reviewer. This protocol has been produced in accordance with the Preferred Reporting Items for Systematic Reviews and Meta-analyses Protocol (PRISMA-P) guidelines.

Ethics and dissemination: As a review of previously published studies, no ethical approval is required. The results of this review will be submitted to a peer reviewed journal for publication. The results can be used in future research into respiratory symptoms that uses electronic healthcare databases.

Registration: This protocol has been registered with PROSPERO with registration number: CRD42018100830

For peer review only

Article Summary

Strengths and Limitations

- To our knowledge, this is the first systematic review to identify codelists for respiratory symptoms used in electronic health records research.
- The results will allow future researchers of respiratory symptoms to reuse or build on already established codelists, which is particularly relevant to health services research that may be more concerned with symptom presentation than diagnosis.
- Due to poor reporting of codelists in published literature, we may identify studies that have used a codelist without including further information about it in either their main paper or in supplementary material.
- Codelists derived from different databases and coding classifications may be difficult to compare and this heterogeneity may limit our ability to synthesise results that are generalisable.

For peer review only

Introduction

Respiratory illnesses such as asthma and chronic obstructive pulmonary disease (COPD) result in significant morbidity worldwide(1). There is also known to be a large degree of under diagnosis, as these conditions are common and the initial symptoms are mild, such that patients may not present to primary care or receive a definitive diagnosis in the early stages of disease(2). The most common symptoms associated with these conditions include breathlessness, wheeze, coughing and sputum production(3,4). These symptoms are relatively non-specific, being associated with other medical conditions such as heart failure or pneumonia, which creates further diagnostic challenges(5). Thus, research into symptom presentation is important, as it could capture those patients presenting for care who may not have received a formal diagnosis.

The widespread use of electronic health records (EHRs) in healthcare has resulted in the creation of large population-based databases, which present unique opportunities for research(6). Research using EHRs typically begins with the extraction of structured data: clinical symptoms, diagnoses and treatments which have been recorded in databases as clinical codes(7). Given the range of codes and variations included in classification systems, a codelist is required to define the computable phenotype and comprehensively capture all patients with a variable of interest(8,9). For more complex clinical concepts, logical expressions may be needed to combine codes; but for simpler concepts, a simple list of relevant codes may be used. Defining this list of codes is a key early step in the research process, as the omission of important codes and inclusion of inappropriate codes can lead to selection biases, affecting the numbers and types of patients captured for study, which in turn impacts on the validity of research findings(7,10). However, as EHR research is a relatively new field, codelists are often poorly reported, as are the methods used to generate them(10).

In recent years, best practice guidelines have begun to be established to improve the validity and reproducibility of research using EHRs(11,12). For example, methods have been developed describing how to construct codelists in a more systematic and reproducible way(13) and online repositories have been developed to aid in the sharing of codelists(14). A recent review identified different methods of codelist development, such as: creating a list of synonyms for the condition of interest; use of hierarchies in coding classification systems; reviewing code sets with clinician input; and updating existing code sets(8). The publication of codelists, and the process of their construction, ensures that research conducted using EHRs is transparent, reproducible and can be scrutinised.

Previous studies have aimed to establish phenotyping methodologies and validation for conditions such as asthma(15) and COPD(16,17). However, to our knowledge, there is currently no current consensus or systematic review around code-lists for respiratory symptoms. This is particularly

For peer review only

relevant for health services research and system planning, which may be more concerned with patient presentation than formal diagnosis. A review of existing codelists for respiratory symptoms would also be useful for future researchers, potentially allowing them to reuse or build on already established codelists.

Methods and Analysis

The primary objective of this review is to develop understanding of the codelists used for identifying respiratory symptoms in EHR research. Relevant questions include:

1. What codelists have been developed for looking at respiratory symptoms from EHRs?
2. How do these codelists compare in terms of the type, length and breadth of codes included?
3. How are codelists combined to define specific symptoms?

Search Strategy

A comprehensive search of the MEDLINE and EMBASE databases (via the HDAS interface) will be conducted. A search strategy has been developed using a combination of keywords and MeSH (Medical Subject Heading) terms relating to 1) electronic health records, 2) codelists, 3) respiratory symptoms, including synonyms for these terms. The full search strategy with terms used can be found in Supplementary File 1. Additionally, the reference lists of articles found will be reviewed for other potential studies, and where the codelist itself is not fully reported in a paper, we will contact the authors of the paper to obtain this. In addition, the following codelist repositories will be searched for information existing outside of the peer-reviewed literature: ClinicalCodes.org(18), CALIBERcodelists(19), CPRD@Cambridge Codelists(20), Phenotype Knowledge Base(21), and the NIH Value Set Authority Centre(22). The study screening process will be recorded using the PRISMA flow diagram(23).

Eligibility Criteria

The inclusion criteria are: studies looking at respiratory symptoms (specifically: cough, shortness of breath, wheeze and sputum) that are based on research using routine data from electronic health records, and that describe use of a codelist (or equivalent). All potential coding classification systems will be eligible. Studies in the English language from any country and published between January 1990 and January 2018 will be included. Exclusion criteria are: studies with codelists for respiratory diagnoses only, rather than symptoms, and studies based on primary data collection rather than secondary data analysis of routine data from electronic databases.

For peer review only

Data Collection

Identified studies will be stored using Rayyan systematic review software(24), and duplicate studies will be removed. Two investigators will separately assess the title and abstracts against the eligibility criteria to screen for papers for inclusion. For papers where either reviewer is unclear whether it meets the eligibility criteria, the full paper will be screened. Eligible papers will then have full text screened, and disagreements about inclusion will be resolved by discussion with a third reviewer to reach a consensus. Key data from each included study will be extracted and recorded in a uniform table using Microsoft Excel. The key data variables that will be extracted are: Study details (title, first author, year of publication); Population (country, time period, target population); Research question or area of interest; Target symptom or symptoms; Data source (type of healthcare database); Code classification system; Code list; Coding algorithm; method used to develop codelist. The codelists are the main data item of interest, and they will be summarised and tabulated to facilitate cross-comparison of the codes included in each list. A narrative synthesis(25) will be conducted to describe differences in the code lists.

Bias

As individual study outcomes are not of interest in this systematic review, we will not consider risk of bias in individual studies. A publication bias may exist as there may be studies using codelists that are not identified as they have not been published in the literature (for example, studies that did not have positive findings) though this will not be formally assessed.

We used the PRISMA-P checklist(26) when writing this protocol (see Supplementary File 2).

Patient and Public Involvement

Patients and the public were not involved in the development of this protocol.

Ethics and Dissemination

This review synthesises information that is already in the public domain, and does not directly involve human participants, and so ethical approval is not required. The protocol has been registered with the PROSPERO International Prospective Register of Systematic Reviews, with registration number…Findings of the review will be disseminated via presentation at relevant conferences and publication in a peer-reviewed journal.

For peer review only

References

1. Ferkol T, Schraufnagel D. The Global Burden of Respiratory Disease. Ann Am Thorac Soc. 2014 Mar 1;11(3):404–6.

2. Bernd L, Joan BS, Michael S, Bernhard K, Lowie EV, Louisa G, et al. Determinants of underdiagnosis of COPD in national and international surveys. Chest. 2015 Oct;148(4):971–85.

3. Chronic obstructive pulmonary disease (COPD) [Internet]. World Health Organization. [cited 2018 May 18]. Available from: http://www.who.int/news-room/fact-sheets/detail/chronic-obstructive-pulmonary-disease-(copd)

4. Asthma [Internet]. World Health Organization. [cited 2018 May 18]. Available from: http://www.who.int/news-room/fact-sheets/detail/asthma

5. Hawkins NM, Petrie MC, Jhund PS, Chalmers GW, Dunn FG, McMurray JJV. Heart failure and chronic obstructive pulmonary disease: diagnostic pitfalls and epidemiology. Eur J Heart Fail. 2009 Feb;11(2):130–9.

6. Casey JA, Schwartz BS, Stewart WF, Adler NE. Using Electronic Health Records for Population Health Research: A Review of Methods and Applications. Annu Rev Public Health. 2016;37(1):61–81.

7. Hripcsak G, Albers DJ. Next-generation phenotyping of electronic health records. J Am Med Inform Assoc. 2013 Jan 1;20(1):117–21.

8. Williams R, Kontopantelis E, Buchan I, Peek N. Clinical code set engineering for reusing EHR data for research: A review. J Biomed Inform. 2017 Jun 1;70:1–13.

9. Richesson R, Smerek M, Zozus N, Chauduri P, Califf R, Simon G, et al. Electronic Health Records-Based Phenotyping, Rethinking Clinical Trials [Internet]. 2014 [cited 2018 Jul 26]. Available from: http://rethinkingclinicaltrials.org/resources/ehr-phenotyping/

10. Herrett E, Thomas S, Schoonen M, Smeeth L, Hall A. Validation and validity of diagnoses in the General Practice Research Database: a systematic review. Br J Clin Pharmacol. 2009 Dec 23;69(1):4–14.

11. Ehrenstein V, Petersen I, Smeeth L, Jick SS, Benchimol EI, Ludvigsson JF, et al. Helping everyone do better: a call for validation studies of routinely recorded health data [Internet]. Clinical Epidemiology. 2016 [cited 2018 Apr 23]. Available from: https://www.dovepress.com/helping-everyone-do-better-a-call-for-validation-studies-of-routinely--peer-reviewed-article-CLEP

12. Denaxas S, Direk K, Gonzalez-Izquierdo A, Pikoula M, Cakiroglu A, Moore J, et al. Methods for enhancing the reproducibility of biomedical research findings using electronic health records. BioData Min. 2017 Sep 11;10:31.

13. Davé S, Petersen I. Creating medical and drug code lists to identify cases in primary care databases. Pharmacoepidemiol Drug Saf. 2009 Aug;18(8):704–7.

14. Springate DA, Kontopantelis E, Ashcroft DM, Olier I, Parisi R, Chamapiwa E, et al. ClinicalCodes: An Online Clinical Codes Repository to Improve the Validity and Reproducibility of Research Using Electronic Medical Records. PLOS ONE. 2014 Jun 18;9(6):e99825.

For peer review only

15. Nissen F, Quint J, Wilkinson S, Müllerova H, Smeeth L, Douglas IJ. Validation of asthma recording in electronic health records: a systematic review. Clin Epidemiol. 2017 Dec;Volume 9:643–56.

16. Rothnie KJ, Müllerová H, Hurst JR, Smeeth L, Davis K, Thomas SL, et al. Validation of the Recording of Acute Exacerbations of COPD in UK Primary Care Electronic Healthcare Records., Validation of the Recording of Acute Exacerbations of COPD in UK Primary Care Electronic Healthcare Records. PloS One PLoS ONE. 2016;11, 11(3, 3):e0151357–e0151357.

17. Rimland JM, Abraha I, Luchetta ML, Cozzolino F, Orso M, Cherubini A, et al. Validation of chronic obstructive pulmonary disease (COPD) diagnoses in healthcare databases: a systematic review protocol. BMJ Open. 2016 Jun 1;6(6):e011777.

18. ClinicalCodes Repository [Internet]. [cited 2018 Jul 5]. Available from: https://clinicalcodes.rss.mhs.man.ac.uk/

19. R-Forge: CALIBER health records research toolkit: R Development Page [Internet]. [cited 2018 Jul 5]. Available from: http://r-forge.r-project.org/R/?group_id=1598

20. CPRD @ Cambridge - Code Lists [Internet]. Primary Care Unit. [cited 2018 Jul 5]. Available from: http://www.phpc.cam.ac.uk/pcu/cprd_cam/codelists/

21. What is the Phenotype KnowledgeBase? | PheKB [Internet]. [cited 2018 Jul 11]. Available from: https://phekb.org/

22. Value Set Authority Center [Internet]. [cited 2018 Jul 11]. Available from: https://vsac.nlm.nih.gov/

23. Moher D, Liberati A, Tetzlaff J, Altman DG, Group TP. Preferred Reporting Items for Systematic Reviews and Meta-Analyses: The PRISMA Statement. PLOS Med. 2009 Jul 21;6(7):e1000097.

24. Ouzzani M, Hammady H, Fedorowicz Z, Elmagarmid A. Rayyan—a web and mobile app for systematic reviews. Syst Rev [Internet]. 2016 Dec [cited 2018 Jul 5];5(1). Available from: http://systematicreviewsjournal.biomedcentral.com/articles/10.1186/s13643-016-0384-4

25. Popay J, Roberts H, Sowden A, Petticrew M, Arai L, Rodgers M, et al. Guidance on the Conduct of Narrative Synthesis in Systematic Review: A Product from the ESRC Methods Programmes [Internet]. Available from: http://citeseerx.ist.psu.edu/viewdoc/download?doi=10.1.1.178.3100&rep=rep1&type=pdf

26. Moher D, Shamseer L, Clarke M, Ghersi D, Liberati A, Petticrew M, et al. Preferred reporting items for systematic review and meta-analysis protocols (PRISMA-P) 2015 statement. Syst Rev. 2015 Jan 1;4(1):1.

For peer review only

Acknowledgements

None

Author Statement

JG and JQ developed the research question. WJ summarised the literature and drafted the protocol, with input into the methodology, search strategy and manuscript from PS, JQ, JG and RA. All authors read, commented on and approved the final manuscript. Any amendments or updates to this protocol will be documented by WJ. Correspondence to WJ.

Funding

JG is funded by a Health Education England / National Institute of Health Research Clinical Lectureship (ICA-CL-2016-02-024). RWA is supported by a Wellcome Trust Clinical Research Career Development Fellowship (206602/Z/17/Z). Funders had no role or input in this protocol.

Conflicts of Interests

All authors report no known conflicts of interest in this work

For peer review only

HDAS Export
Search Strategy EMBASE

11 Jul 18 - 13:44

Strategy 463864

#	Database	Search term	Results
1	EMBASE	exp "MEDICAL RECORD"/ OR "ELECTRONIC HEALTH RECORDS"/ OR "ELECTRONIC HEALTHCARE DATABASE"/ OR "ELECTRONIC MEDICAL RECORD SYSTEM"/	211275
2	EMBASE	(EHR OR "Electronic Health Record*" OR ((electr* OR digit* OR computer* OR linked OR administrative OR routine OR personal OR patient OR health OR medical) AND (record* OR database*))).ti,ab,if	932977
3	EMBASE	(1 OR 2)	1012318
4	EMBASE	CODING/ OR exp "PATIENT CODING"/ OR "CODING ALGORITHM"/	26988
5	EMBASE	(code OR codes OR coding OR codelist* OR codeset* OR "value set" OR phenotyp*).ti,ab,if	953908
6	EMBASE	(4 OR 5)	974209
7	EMBASE	exp COUGHING/	104032
8	EMBASE	exp WHEEZING/	23552
9	EMBASE	exp SPUTUM/	22594
10	EMBASE	exp DYSPNEA/ OR exp DYSPNOEA/	143174
11	EMBASE	("respirat* symptom*" OR (respirat* AND symptom*) OR breath* OR dyspn* OR sputum OR phlegm OR cough* OR wheez*).ti,ab,if	375955
12	EMBASE	(7 OR 8 OR 9 OR 10 OR 11)	491214
13	EMBASE	(3 AND 6 AND 12)	1108
14	EMBASE	3 AND 6 AND 12 [DT 1990-2017] [English language]	1005

HDAS Export
Search Strategy MEDLINE

11 Jul 18 - 13:50

Strategy 463861

#	Database	Search term	Results
1	Medline	exp "MEDICAL RECORDS"/ OR "HEALTH RECORDS, PERSONAL"/ OR "MEDICAL RECORD LINKAGE"/ OR "MEDICAL RECORDS SYSTEMS, COMPUTERISED"/ OR "ELECTRONIC HEALTH RECORDS"/	134717
2	Medline	(EHR OR "Electronic Health Record*" OR EMR OR "Electronic Medical Record*" OR ((electr* OR digit* OR computer* OR linked OR administrative OR routine OR personal OR patient OR medical OR health) AND (record* OR database*))).ti,ab,af	909067
3	Medline	(1 OR 2)	937921
4	Medline	exp "CLINICAL CODING"/	1753
5	Medline	(code OR codes OR coding OR codelist* OR codeset* OR "value set" OR phenotyp*).ti,ab,af	821866
6	Medline	(4 OR 5)	821866
7	Medline	exp COUGH/	14517
8	Medline	exp "SIGNS AND SYMPTOMS, RESPIRATORY"/	165589
9	Medline	exp DYSPNEA/	19188
10	Medline	exp SPUTUM/	19961
11	Medline	("respirat* symptom*" OR (respirat* AND symptom*) OR breath* OR dyspn* OR sputum OR phlegm OR cough* OR wheez*).ti,ab,af	293228
12	Medline	(7 OR 8 OR 9 OR 10 OR 11)	401014
13	Medline	(3 AND 6 AND 12)	620
14	Medline	3 AND 6 AND 12 [DT 1990-2017] [Languages English]	550

Reporting checklist for protocol of a systematic review.

Based on the PRISMA-P guidelines.

Instructions to authors

Complete this checklist by entering the page numbers from your manuscript where readers will find each of the items listed below.

Your article may not currently address all the items on the checklist. Please modify your text to include the missing information. If you are certain that an item does not apply, please write "n/a" and provide a short explanation.

Upload your completed checklist as an extra file when you submit to a journal.

In your methods section, say that you used the PRISMA-P reporting guidelines, and cite them as:

Moher D, Shamseer L, Clarke M, Ghersi D, Liberati A, Petticrew M, Shekelle P, Stewart LA. Preferred Reporting Items for Systematic Review and Meta-Analysis Protocols (PRISMA-P) 2015 statement. Syst Rev. 2015;4(1):1.

		Reporting Item	Page Number
Identification	#1a	Identify the report as a protocol of a systematic review	1
Update	#1b	If the protocol is for an update of a previous systematic review, identify as such	See note 1
	#2	If registered, provide the name of the registry (such as PROSPERO) and registration number	2
Contact	#3a	Provide name, institutional affiliation, e-mail address of all protocol authors; provide physical mailing address of corresponding author	1
Contribution	#3b	Describe contributions of protocol authors and identify the guarantor of the review	9
	#4	If the protocol represents an amendment of a previously completed or published protocol, identify as such and list changes; otherwise, state plan for documenting important	9

For peer review only

		protocol amendments	
Sources	#5a	Indicate sources of financial or other support for the review	9
Sponsor	#5b	Provide name for the review funder and / or sponsor	9
Role of sponsor or funder	#5c	Describe roles of funder(s), sponsor(s), and / or institution(s), if any, in developing the protocol	9
Rationale	#6	Describe the rationale for the review in the context of what is already known	4
Objectives	#7	Provide an explicit statement of the question(s) the review will address with reference to participants, interventions, comparators, and outcomes (PICO)	5
Eligibility criteria	#8	Specify the study characteristics (such as PICO, study design, setting, time frame) and report characteristics (such as years considered, language, publication status) to be used as criteria for eligibility for the review	5
Information sources	#9	Describe all intended information sources (such as electronic databases, contact with study authors, trial registers or other grey literature sources) with planned dates of coverage	5
Search strategy	#10	Present draft of search strategy to be used for at least one electronic database, including planned limits, such that it could be repeated	5
Study records - data management	#11a	Describe the mechanism(s) that will be used to manage records and data throughout the review	6
Study records - selection process	#11b	State the process that will be used for selecting studies (such as two independent reviewers) through each phase of the review (that is, screening, eligibility and inclusion in meta-analysis)	6
Study records - data collection process	#11c	Describe planned method of extracting data from reports (such as piloting forms, done independently, in duplicate), any processes for obtaining and confirming data from investigators	6
Data items	#12	List and define all variables for which data will be sought (such as PICO items, funding sources), any pre-planned data assumptions and simplifications	6

For peer review only

Outcomes and prioritization	#13	List and define all outcomes for which data will be sought, including prioritization of main and additional outcomes, with rationale	6
Risk of bias in individual studies	#14	Describe anticipated methods for assessing risk of bias of individual studies, including whether this will be done at the outcome or study level, or both; state how this information will be used in data synthesis	6
Data synthesis	#15a	Describe criteria under which study data will be quantitatively synthesised	See note 2
	#15b	If data are appropriate for quantitative synthesis, describe planned summary measures, methods of handling data and methods of combining data from studies, including any planned exploration of consistency (such as I2, Kendall's τ)	See note 3
	#15c	Describe any proposed additional analyses (such as sensitivity or subgroup analyses, meta-regression)	See note 4
	#15d	If quantitative synthesis is not appropriate, describe the type of summary planned	6
Meta-bias(es)	#16	Specify any planned assessment of meta-bias(es) (such as publication bias across studies, selective reporting within studies)	6
Confidence in cumulative evidence	#17	Describe how the strength of the body of evidence will be assessed (such as GRADE)	See note 5

Author notes

1. N/A - This is not an update
2. N/A - No quantitative synthesis of findings will be conducted
3. N/A - No quantitative synthesis of findings will be conducted
4. N/A - No quantitative synthesis of findings will be conducted
5. N/A - Study outcomes from individual studies are not the object of interest

For peer review only

The PRISMA-P checklist is distributed under the terms of the Creative Commons Attribution License CC-BY 4.0. This checklist was completed on 06. August 2018 using http://www.goodreports.org/, a tool made by the EQUATOR Network in collaboration with Penelope.ai

For peer review only

Code sets for respiratory symptoms in electronic health records (EHR) research: a systematic review protocol

Journal:	*BMJ Open*
Manuscript ID	bmjopen-2018-025965.R1
Article Type:	Protocol
Date Submitted by the Author:	18-Jan-2019
Complete List of Authors:	Jayatunga, Wikum; University College London, Institute of Health Informatics<br>Stone, Philip; Imperial College London, National Heart and Lung Institute<br>Aldridge, Robert; University College London, Institute of Health Informatics<br>Quint, Jennifer; Imperial College London, Respiratory Epidemiology, Occupational Medicine and Public Health<br>George, Julie; University College London, Institute of Health Informatics
**Primary Subject Heading**:	Health informatics
Secondary Subject Heading:	Health informatics, Respiratory medicine, Health services research
Keywords:	Electronic Health Records, Coding, Clinical Coding, Respiratory, Symptoms, Code sets

SCHOLARONE™ Manuscripts

Title

Code sets for respiratory symptoms in electronic health records (EHR) research: a systematic review protocol

Authors

Dr Wikum Jayatunga[a], wikumj@gmail.com
Mr Philip Stone[b], p.stone@imperial.ac.uk
Dr Robert W Aldridge[a], r.aldridge@ucl.ac.uk
Dr Jennifer K Quint[b], j.quint@imperial.ac.uk
Dr Julie George[a], j.george@ucl.ac.uk

[a] University College London, Institute of Health Informatics, 222 Euston Road, London NW1 2DA
[b] Imperial College London, National Heart and Lung Institute, 1B Manresa Road, London, SW3 6LR

Corresponding Author
Dr Wikum Jayatunga
Address: 3 Constable Mews, Bromley, BR1 3BF
Email: wikumj@gmail.com
Telephone: 07872959152

Keywords

Electronic Health Records, Coding, Clinical Coding, Respiratory, Symptoms, Code sets, Codelists

Wordcount

Word count = 1417
(excluding title page, article summary, abstract, references, figures and declarations)

For peer review only

Abstract

Introduction: Asthma and chronic obstructive pulmonary disease (COPD) are common respiratory conditions which result in significant morbidity worldwide. These conditions are associated with a range of non-specific symptoms, which in themselves are a target for health research. Such research is increasingly being conducted using electronic health records (EHRs), but computable phenotype definitions, in the form of code sets or codelists, are required to extract structured data from these large routine databases in a systematic and reproducible way. The aim of this protocol is to specify a systematic review to identify code sets for respiratory symptoms in electronic health records research.

Methods and Analysis: MEDLINE and EMBASE databases will be searched using terms relating to electronic health records, respiratory symptoms and use of code sets. The search will cover all English-language studies in these databases between January 1990 and December 2017. Two reviewers will independently screen identified studies for inclusion, and key data will be extracted into a uniform table, facilitating cross-comparison of codes used. Disagreements between the reviewers will be adjudicated by a third reviewer. This protocol has been produced in accordance with the Preferred Reporting Items for Systematic Reviews and Meta-analyses Protocol (PRISMA-P) guidelines.

Ethics and dissemination: As a review of previously published studies, no ethical approval is required. The results of this review will be submitted to a peer reviewed journal for publication. The results can be used in future research into respiratory symptoms that uses electronic healthcare databases.

Registration: This protocol has been registered with PROSPERO with registration number: CRD42018100830

For peer review only

Article Summary

Strengths and Limitations

- To our knowledge, this is the first systematic review to identify code sets for respiratory symptoms used in electronic health records research.

- The results will allow future researchers of respiratory symptoms to reuse or build on already established code sets, which is particularly relevant to health services research that may be more concerned with symptom presentation than diagnosis.

- Due to poor reporting of code sets in published literature, we may identify studies that have used a code set without including further information about it in either their main paper or in supplementary material.

- Code sets derived from different databases and coding classifications may be difficult to compare and this heterogeneity may limit our ability to synthesise results that are generalisable.

For peer review only

Introduction

Respiratory illnesses such as asthma and chronic obstructive pulmonary disease (COPD) result in significant morbidity worldwide(1). There is also known to be a large degree of under diagnosis, as these conditions are common and the initial symptoms are mild, such that patients may not present to primary care or receive a definitive diagnosis in the early stages of disease(2). The most common symptoms associated with these conditions include breathlessness, wheeze, coughing and sputum production(3,4). These symptoms are relatively non-specific, being associated with other medical conditions such as heart failure or pneumonia, which creates further diagnostic challenges(5). Thus, research into symptom presentation is important, as it could capture those patients presenting for care who may not have received a formal diagnosis.

The widespread use of electronic health records (EHRs) in healthcare has resulted in the creation of large population-based databases, which present unique opportunities for research(6). Research using EHRs typically begins with the extraction of structured data: clinical symptoms, diagnoses and treatments which have been recorded in databases as clinical codes(7). Given the range and variety of codes included in classification systems, a collection or list of unique values ('code set' or 'code list') can be used to identify a particular clinical concept. These code sets can be used, sometimes alongside other data such as medications and test results, to define the computable phenotype and comprehensively capture all patients with a variable of interest(8,9). For more complex clinical concepts, logical expressions may be needed to combine codes; but for simpler concepts, a simple list of relevant codes may be used. Defining this list of codes is a key early step in the research process, as the omission of important codes and inclusion of inappropriate codes can lead to selection biases, affecting the numbers and types of patients captured for study, which in turn impacts on the validity of research findings(7,10). However, as EHR research is a relatively new field, code sets are often poorly reported in published studies, as are the methods used to generate them(10).

In recent years, best practice guidelines have begun to be established to improve the validity and reproducibility of research using EHRs(11,12). For example, methods have been developed describing how to construct code sets in a more systematic and reproducible way(13,14) and online repositories have been developed to aid in the sharing of code sets(15). A recent review identified different methods of code set development, such as: creating a list of synonyms for the condition of interest; use of hierarchies in coding classification systems; reviewing code sets with clinician input; and updating existing code sets(8). The publication of code sets, and the process of their construction, ensures that research conducted using EHRs is transparent, reproducible and can be scrutinised.

For peer review only

Previous studies have aimed to establish phenotyping methodologies and validation for conditions such as asthma(16) and COPD(17,18). However, to our knowledge, there is currently no current consensus or systematic review around code-lists for respiratory symptoms. This is particularly relevant for health services research and system planning, which may be more concerned with patient presentation than formal diagnosis. A review of existing code sets for respiratory symptoms would also be useful for future researchers, potentially allowing them to reuse or build on already established code sets.

Methods and Analysis

The primary objective of this review is to develop understanding of the code sets used for identifying respiratory symptoms in EHR research. Relevant questions include:

1. What code sets have been developed for looking at respiratory symptoms from EHRs?
2. How do these code sets compare in terms of the type, length and breadth of codes included?
3. How are code sets combined to define particular symptoms?

Search Strategy

A comprehensive search of the MEDLINE and EMBASE databases (via the HDAS interface) will be conducted. A search strategy has been developed using a combination of keywords and MeSH (Medical Subject Heading) terms relating to 1) electronic health records, 2) code sets, 3) respiratory symptoms, including synonyms for these terms. The full search strategy with terms used can be found in Supplementary File 1. Additionally, the reference lists of articles found will be reviewed for other potential studies, and where the code set itself is not fully reported in a paper, we will contact the authors of the paper to obtain this. In addition, the following code set repositories will be searched for information existing outside of the peer-reviewed literature: ClinicalCodes.org(19), CALIBERcodelists(20), CPRD@Cambridge Codelists(21), Phenotype Knowledge Base(22), the NIH Value Set Authority Centre(23), and the LSHTM Data Compass(24). The study screening process will be recorded using the PRISMA flow diagram(25).

Eligibility Criteria

The inclusion criteria are: studies looking at respiratory symptoms (specifically: cough, shortness of breath, wheeze and sputum) that are based on research using routine data from electronic health records, and that describe use of a code set (or equivalent). Broader search terms for 'respiratory symptoms' are included in the search strategy to improve its sensitivity, but after full text screening, only papers relating to the respiratory symptoms above will be included. All potential coding classification systems will be eligible. Studies in the English language from any country and published between 1st January 1990 and 31st December 2017 will be included. Exclusion criteria are: studies

For peer review only

with code sets for respiratory diagnoses only, rather than symptoms, and studies based on primary data collection rather than secondary data analysis of routine data from electronic databases.

Data Collection

Identified studies will be stored using Rayyan systematic review software(26), and duplicate studies will be removed. Two investigators will separately assess the title and abstracts against the eligibility criteria to screen for papers for inclusion. For papers where either reviewer is unclear whether it meets the eligibility criteria, the full paper will be screened. Eligible papers will then have full text screened, and disagreements about inclusion will be resolved by discussion with a third reviewer to reach a consensus. Key data from each included study will be extracted and recorded in a uniform table using Microsoft Excel. The key data variables that will be extracted are: Study details (title, first author, year of publication); Population (country, time period, target population); Research question or area of interest; Target symptom or symptoms; Data source (type of healthcare database); Code classification system; Version of the code classification system; Code set; Coding algorithm for symptom ascertainment; Method used to develop code set. The quality of code set development methodology will be scored using methods established in the literature as a benchmark(13,14). The code sets are the main data item of interest, and they will be summarised and tabulated to facilitate cross-comparison of the codes included in each list. A narrative synthesis(27) will be conducted to describe differences in the code sets.

Limitations

It is known that code sets are poorly reported in the published literature(10). Therefore, we may identify studies that appear to have used a code set but then do not report further information about how it was developed or sourced. Furthermore, including a search term relating to 'code sets' may not pick up papers which have not reported the code set development in their main paper or relegated this information to an appendix. However, including this search term was necessary to improve the specificity of the search. Another limitation is that code sets derived from different systems and using different classification systems may limit our ability for cross-comparison and synthesis of results. Finally, as this is a retrospective review of published code sets, the results may become outdated as clinical terminologies are updated over time, and so researchers using the results from this review to inform their work should be aware of this and conduct additional searches for any new clinical codes of relevance.

Bias

As individual study outcomes are not of interest in this systematic review, we will not consider risk of bias in individual studies. A publication bias may exist as there may be studies using code sets that are not identified as they have not been published in the literature (for example, studies that did not have positive findings) though this will not be formally assessed.

We used the PRISMA-P checklist(28) when writing this protocol (see Supplementary File 2).

For peer review only

Patient and Public Involvement

Patients and the public were not involved in the development of this protocol.

Ethics and Dissemination

This review synthesises information that is already in the public domain, and does not directly involve human participants, and so ethical approval is not required. The protocol has been registered with the PROSPERO International Prospective Register of Systematic Reviews, with registration number CRD42018100830. Findings of the review will be disseminated via presentation at relevant conferences and publication in a peer-reviewed journal.

For peer review only

References

1. Ferkol T, Schraufnagel D. The Global Burden of Respiratory Disease. Ann Am Thorac Soc. 2014 Mar 1;11(3):404–6.

2. Bernd L, Joan BS, Michael S, Bernhard K, Lowie EV, Louisa G, et al. Determinants of underdiagnosis of COPD in national and international surveys. Chest. 2015 Oct;148(4):971–85.

3. Chronic obstructive pulmonary disease (COPD) [Internet]. World Health Organization. [cited 2018 May 18]. Available from: http://www.who.int/news-room/fact-sheets/detail/chronic-obstructive-pulmonary-disease-(copd)

4. Asthma [Internet]. World Health Organization. [cited 2018 May 18]. Available from: http://www.who.int/news-room/fact-sheets/detail/asthma

5. Hawkins NM, Petrie MC, Jhund PS, Chalmers GW, Dunn FG, McMurray JJV. Heart failure and chronic obstructive pulmonary disease: diagnostic pitfalls and epidemiology. Eur J Heart Fail. 2009 Feb;11(2):130–9.

6. Casey JA, Schwartz BS, Stewart WF, Adler NE. Using Electronic Health Records for Population Health Research: A Review of Methods and Applications. Annu Rev Public Health. 2016;37(1):61–81.

7. Hripcsak G, Albers DJ. Next-generation phenotyping of electronic health records. J Am Med Inform Assoc. 2013 Jan 1;20(1):117–21.

8. Williams R, Kontopantelis E, Buchan I, Peek N. Clinical code set engineering for reusing EHR data for research: A review. J Biomed Inform. 2017 Jun 1;70:1–13.

9. Richesson R, Smerek M, Zozus N, Chauduri P, Califf R, Simon G, et al. Electronic Health Records-Based Phenotyping, Rethinking Clinical Trials [Internet]. 2014 [cited 2018 Jul 26]. Available from: http://rethinkingclinicaltrials.org/resources/ehr-phenotyping/

10. Herrett E, Thomas S, Schoonen M, Smeeth L, Hall A. Validation and validity of diagnoses in the General Practice Research Database: a systematic review. Br J Clin Pharmacol. 2009 Dec 23;69(1):4–14.

11. Ehrenstein V, Petersen I, Smeeth L, Jick SS, Benchimol EI, Ludvigsson JF, et al. Helping everyone do better: a call for validation studies of routinely recorded health data [Internet]. Clinical Epidemiology. 2016 [cited 2018 Apr 23]. Available from: https://www.dovepress.com/helping-everyone-do-better-a-call-for-validation-studies-of-routinely--peer-reviewed-article-CLEP

12. Denaxas S, Direk K, Gonzalez-Izquierdo A, Pikoula M, Cakiroglu A, Moore J, et al. Methods for enhancing the reproducibility of biomedical research findings using electronic health records. BioData Min. 2017 Sep 11;10:31.

13. Davé S, Petersen I. Creating medical and drug code lists to identify cases in primary care databases. Pharmacoepidemiol Drug Saf. 2009 Aug;18(8):704–7.

14. Watson J, Nicholson BD, Hamilton W, Price S. Identifying clinical features in primary care electronic health record studies: methods for codelist development. BMJ Open. 2017 Nov 1;7(11):e019637.

For peer review only

15. Springate DA, Kontopantelis E, Ashcroft DM, Olier I, Parisi R, Chamapiwa E, et al. ClinicalCodes: An Online Clinical Codes Repository to Improve the Validity and Reproducibility of Research Using Electronic Medical Records. PLOS ONE. 2014 Jun 18;9(6):e99825.

16. Nissen F, Quint J, Wilkinson S, Müllerova H, Smeeth L, Douglas IJ. Validation of asthma recording in electronic health records: a systematic review. Clin Epidemiol. 2017 Dec;Volume 9:643–56.

17. Rothnie KJ, Müllerová H, Hurst JR, Smeeth L, Davis K, Thomas SL, et al. Validation of the Recording of Acute Exacerbations of COPD in UK Primary Care Electronic Healthcare Records., Validation of the Recording of Acute Exacerbations of COPD in UK Primary Care Electronic Healthcare Records. PloS One PLoS ONE. 2016;11, 11(3, 3):e0151357–e0151357.

18. Rimland JM, Abraha I, Luchetta ML, Cozzolino F, Orso M, Cherubini A, et al. Validation of chronic obstructive pulmonary disease (COPD) diagnoses in healthcare databases: a systematic review protocol. BMJ Open. 2016 Jun 1;6(6):e011777.

19. ClinicalCodes Repository [Internet]. [cited 2018 Jul 5]. Available from: https://clinicalcodes.rss.mhs.man.ac.uk/

20. R-Forge: CALIBER health records research toolkit: R Development Page [Internet]. [cited 2018 Jul 5]. Available from: http://r-forge.r-project.org/R/?group_id=1598

21. CPRD @ Cambridge - Code Lists [Internet]. Primary Care Unit. [cited 2018 Jul 5]. Available from: http://www.phpc.cam.ac.uk/pcu/cprd_cam/codelists/

22. What is the Phenotype KnowledgeBase? | PheKB [Internet]. [cited 2018 Jul 11]. Available from: https://phekb.org/

23. Value Set Authority Center [Internet]. [cited 2018 Jul 11]. Available from: https://vsac.nlm.nih.gov/

24. LSHTM Data Compass [Internet]. [cited 2019 Jan 8]. Available from: https://datacompass.lshtm.ac.uk/

25. Moher D, Liberati A, Tetzlaff J, Altman DG, Group TP. Preferred Reporting Items for Systematic Reviews and Meta-Analyses: The PRISMA Statement. PLOS Med. 2009 Jul 21;6(7):e1000097.

26. Ouzzani M, Hammady H, Fedorowicz Z, Elmagarmid A. Rayyan—a web and mobile app for systematic reviews. Syst Rev [Internet]. 2016 Dec [cited 2018 Jul 5];5(1). Available from: http://systematicreviewsjournal.biomedcentral.com/articles/10.1186/s13643-016-0384-4

27. Popay J, Roberts H, Sowden A, Petticrew M, Arai L, Rodgers M, et al. Guidance on the Conduct of Narrative Synthesis in Systematic Review: A Product from the ESRC Methods Programmes [Internet]. Available from: http://citeseerx.ist.psu.edu/viewdoc/download?doi=10.1.1.178.3100&rep=rep1&type=pdf

28. Moher D, Shamseer L, Clarke M, Ghersi D, Liberati A, Petticrew M, et al. Preferred reporting items for systematic review and meta-analysis protocols (PRISMA-P) 2015 statement. Syst Rev. 2015 Jan 1;4(1):1.

For peer review only

Acknowledgements

None

Author Statement

JG and JQ developed the research question. WJ summarised the literature and drafted the protocol, with input into the methodology, search strategy and manuscript from PS, JQ, JG and RA. All authors read, commented on and approved the final manuscript. Any amendments or updates to this protocol will be documented by WJ. Correspondence to WJ.

Funding

JG is funded by a Health Education England / National Institute of Health Research Clinical Lectureship (ICA-CL-2016-02-024). RWA is supported by a Wellcome Trust Clinical Research Career Development Fellowship (206602/Z/17/Z). Funders had no role or input in this protocol.

Conflicts of Interests

All authors report no known conflicts of interest in this work

For peer review only

HDAS Export
Search Strategy EMBASE

11 Jul 18 - 13:44

Strategy 463864

#	Database	Search term	Results
1	EMBASE	exp "MEDICAL RECORD"/ OR "ELECTRONIC HEALTH RECORDS"/ OR "ELECTRONIC HEALTHCARE DATABASE"/ OR "ELECTRONIC MEDICAL RECORD SYSTEM"/	211275
2	EMBASE	(EHR OR "Electronic Health Record*" OR ((electr* OR digit* OR computer* OR linked OR administrative OR routine OR personal OR patient OR health OR medical) AND (record* OR database*))).ti,ab,if	932977
3	EMBASE	(1 OR 2)	1012318
4	EMBASE	CODING/ OR exp "PATIENT CODING"/ OR "CODING ALGORITHM"/	26988
5	EMBASE	(code OR codes OR coding OR codelist* OR codeset* OR "value set" OR phenotyp*).ti,ab,if	953908
6	EMBASE	(4 OR 5)	974209
7	EMBASE	exp COUGHING/	104032
8	EMBASE	exp WHEEZING/	23552
9	EMBASE	exp SPUTUM/	22594
10	EMBASE	exp DYSPNEA/ OR exp DYSPNOEA/	143174
11	EMBASE	("respirat* symptom*" OR (respirat* AND symptom*) OR breath* OR dyspn* OR sputum OR phlegm OR cough* OR wheez*).ti,ab,if	375955
12	EMBASE	(7 OR 8 OR 9 OR 10 OR 11)	491214
13	EMBASE	(3 AND 6 AND 12)	1108
14	EMBASE	3 AND 6 AND 12 [DT 1990-2017] [English language]	1005

Page 1 of 1

HDAS Export

Search Strategy MEDLINE

11 Jul 18 - 13:50

Strategy 463861

#	Database	Search term	Results
1	Medline	exp "MEDICAL RECORDS"/ OR "HEALTH RECORDS, PERSONAL"/ OR "MEDICAL RECORD LINKAGE"/ OR "MEDICAL RECORDS SYSTEMS, COMPUTERISED"/ OR "ELECTRONIC HEALTH RECORDS"/	134717
2	Medline	(EHR OR "Electronic Health Record*" OR EMR OR "Electronic Medical Record*" OR ((electr* OR digit* OR computer* OR linked OR administrative OR routine OR personal OR patient OR medical OR health) AND (record* OR database*))).ti,ab,af	909067
3	Medline	(1 OR 2)	937921
4	Medline	exp "CLINICAL CODING"/	1753
5	Medline	(code OR codes OR coding OR codelist* OR codeset* OR "value set" OR phenotyp*).ti,ab,af	821866
6	Medline	(4 OR 5)	821866
7	Medline	exp COUGH/	14517
8	Medline	exp "SIGNS AND SYMPTOMS, RESPIRATORY"/	165589
9	Medline	exp DYSPNEA/	19188
10	Medline	exp SPUTUM/	19961
11	Medline	("respirat* symptom*" OR (respirat* AND symptom*) OR breath* OR dyspn* OR sputum OR phlegm OR cough* OR wheez*).ti,ab,af	293228
12	Medline	(7 OR 8 OR 9 OR 10 OR 11)	401014
13	Medline	(3 AND 6 AND 12)	620
14	Medline	3 AND 6 AND 12 [DT 1990-2017] [Languages English]	550

Reporting checklist for protocol of a systematic review.

Based on the PRISMA-P guidelines.

Instructions to authors

Complete this checklist by entering the page numbers from your manuscript where readers will find each of the items listed below.

Your article may not currently address all the items on the checklist. Please modify your text to include the missing information. If you are certain that an item does not apply, please write "n/a" and provide a short explanation.

Upload your completed checklist as an extra file when you submit to a journal.

In your methods section, say that you used the PRISMA-P reporting guidelines, and cite them as:

Moher D, Shamseer L, Clarke M, Ghersi D, Liberati A, Petticrew M, Shekelle P, Stewart LA. Preferred Reporting Items for Systematic Review and Meta-Analysis Protocols (PRISMA-P) 2015 statement. Syst Rev. 2015;4(1):1.

		Reporting Item	Page Number
Identification	#1a	Identify the report as a protocol of a systematic review	1
Update	#1b	If the protocol is for an update of a previous systematic review, identify as such	See note 1
	#2	If registered, provide the name of the registry (such as PROSPERO) and registration number	2
Contact	#3a	Provide name, institutional affiliation, e-mail address of all protocol authors; provide physical mailing address of corresponding author	1
Contribution	#3b	Describe contributions of protocol authors and identify the guarantor of the review	9
	#4	If the protocol represents an amendment of a previously completed or published protocol, identify as such and list changes; otherwise, state plan for documenting important	9

For peer review only

		protocol amendments	
Sources	#5a	Indicate sources of financial or other support for the review	9
Sponsor	#5b	Provide name for the review funder and / or sponsor	9
Role of sponsor or funder	#5c	Describe roles of funder(s), sponsor(s), and / or institution(s), if any, in developing the protocol	9
Rationale	#6	Describe the rationale for the review in the context of what is already known	4
Objectives	#7	Provide an explicit statement of the question(s) the review will address with reference to participants, interventions, comparators, and outcomes (PICO)	5
Eligibility criteria	#8	Specify the study characteristics (such as PICO, study design, setting, time frame) and report characteristics (such as years considered, language, publication status) to be used as criteria for eligibility for the review	5
Information sources	#9	Describe all intended information sources (such as electronic databases, contact with study authors, trial registers or other grey literature sources) with planned dates of coverage	5
Search strategy	#10	Present draft of search strategy to be used for at least one electronic database, including planned limits, such that it could be repeated	5
Study records - data management	#11a	Describe the mechanism(s) that will be used to manage records and data throughout the review	6
Study records - selection process	#11b	State the process that will be used for selecting studies (such as two independent reviewers) through each phase of the review (that is, screening, eligibility and inclusion in meta-analysis)	6
Study records - data collection process	#11c	Describe planned method of extracting data from reports (such as piloting forms, done independently, in duplicate), any processes for obtaining and confirming data from investigators	6
Data items	#12	List and define all variables for which data will be sought (such as PICO items, funding sources), any pre-planned data assumptions and simplifications	6

For peer review only

Outcomes and prioritization	#13	List and define all outcomes for which data will be sought, including prioritization of main and additional outcomes, with rationale	6
Risk of bias in individual studies	#14	Describe anticipated methods for assessing risk of bias of individual studies, including whether this will be done at the outcome or study level, or both; state how this information will be used in data synthesis	6
Data synthesis	#15a	Describe criteria under which study data will be quantitatively synthesised	See note 2
	#15b	If data are appropriate for quantitative synthesis, describe planned summary measures, methods of handling data and methods of combining data from studies, including any planned exploration of consistency (such as I2, Kendall's τ)	See note 3
	#15c	Describe any proposed additional analyses (such as sensitivity or subgroup analyses, meta-regression)	See note 4
	#15d	If quantitative synthesis is not appropriate, describe the type of summary planned	6
Meta-bias(es)	#16	Specify any planned assessment of meta-bias(es) (such as publication bias across studies, selective reporting within studies)	6
Confidence in cumulative evidence	#17	Describe how the strength of the body of evidence will be assessed (such as GRADE)	See note 5

For peer review only

Author notes

1. N/A - This is not an update
2. N/A - No quantitative synthesis of findings will be conducted
3. N/A - No quantitative synthesis of findings will be conducted
4. N/A - No quantitative synthesis of findings will be conducted
5. N/A - Study outcomes from individual studies are not the object of interest

The PRISMA-P checklist is distributed under the terms of the Creative Commons Attribution License CC-BY 4.0. This checklist was completed on 06. August 2018 using http://www.goodreports.org/, a tool made by the EQUATOR Network in collaboration with Penelope.ai

For peer review only
